# Importance of the (Pro)renin Receptor in Activating the Renin-Angiotensin System During Normotensive and Preeclamptic Pregnancies

**DOI:** 10.1007/s11906-024-01316-1

**Published:** 2024-08-02

**Authors:** Lachlan G. Schofield, Saije K. Endacott, Sarah J. Delforce, Eugenie R. Lumbers, Kirsty G. Pringle

**Affiliations:** 1 School of Biomedical Sciences and Pharmacy, College of Health, Medicine and Wellbeing, University of Newcastle, Callaghan, N.S.W 2308 Australia; 2 Womens Health Research Program, Hunter Medical Research Institute, New Lambton Heights, N.S.W 2305 Australia; 3 School of Medicine and Public Health, College of Health, Medicine and Wellbeing, University of Newcastle, Callaghan, N.S.W 2308 Australia; 4 Hunter Medical Research Institute, Lot 1 Kookaburra Circuit, New Lambton, N.S.W 2305 Australia

**Keywords:** (Pro)renin Receptor ((P)RR), Preeclampsia, Renin Angiotensin System (RAS)

## Abstract

**Purpose of Review:**

For a healthy pregnancy to occur, a controlled interplay between the maternal circulating renin–angiotensin–aldosterone system (RAAS), placental renin-angiotensin system (RAS) and intrarenal renin-angiotensin system (iRAS) is necessary. Functionally, both the RAAS and iRAS interact to maintain blood pressure and cardiac output, as well as fluid and electrolyte balance. The placental RAS is important for placental development while also influencing the maternal circulating RAAS and iRAS. This narrative review concentrates on the (pro)renin receptor ((P)RR) and its soluble form (s(P)RR) in the context of the hypertensive pregnancy pathology, preeclampsia.

**Recent Findings:**

The (P)RR and the s(P)RR have become of particular interest as not only can they activate prorenin and renin, thus influencing levels of angiotensin II (Ang II), but s(P)RR has now been shown to directly interact with and stimulate the Angiotensin II type 1 receptor (AT_1_R). Levels of both placental (P)RR and maternal circulating s(P)RR are elevated in patients with preeclampsia. Furthermore, s(P)RR has been shown to increase blood pressure in non-pregnant and pregnant rats and mice.

**Summary:**

In preeclamptic pregnancies, which are characterised by maternal hypertension and impaired placental development and function, we propose that there is enhanced secretion of s(P)RR from the placenta into the maternal circulation. Due to its ability to both activate prorenin and act as an AT_1_R agonist, excess maternal circulating s(P)RR can act on both the maternal vasculature, and the kidney, leading to RAS over-activation. This results in dysregulation of the maternal circulating RAAS and overactivation of the iRAS, contributing to maternal hypertension, renal damage, and secondary changes to neurohumoral regulation of fluid and electrolyte balance, ultimately contributing to the pathophysiology of preeclampsia.

## Introduction

The maternal circulating renin-angiotensin aldosterone system (RAAS), placental renin-angiotensin system (RAS) and intrarenal renin-angiotensin system (iRAS) are responsible for significant maternal cardiovascular and renal adaptations throughout pregnancy to meet the needs of the mother, and the growing demands of the conceptus [1–3]. Dysregulation of these renin-angiotensin systems (RASs) and the interplay between them can significantly impact maternal and fetal health, leading to the development of hypertensive disorders of pregnancy [4].

**Fig. 1:**
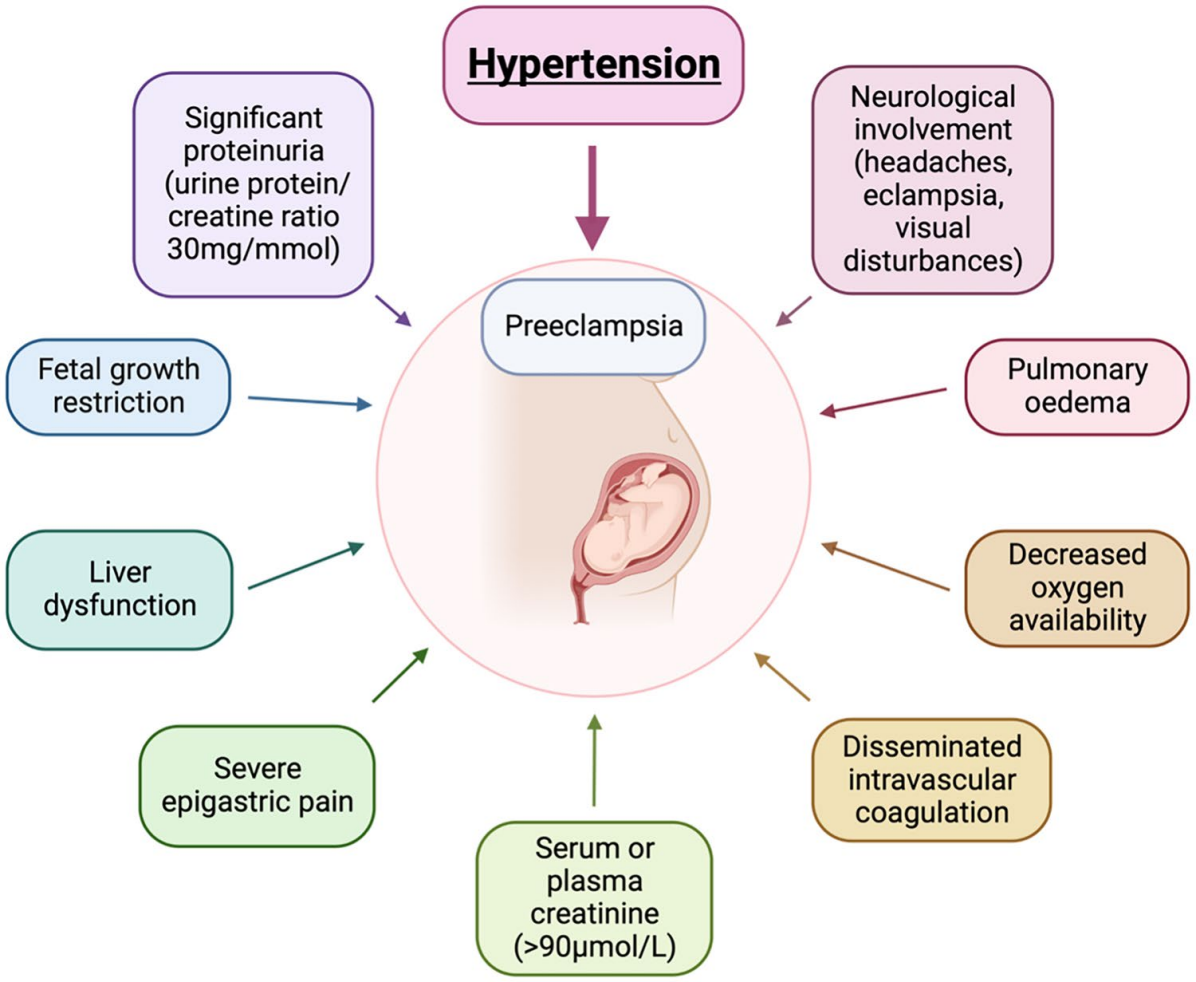
Diagnostic criteria of preeclampsia. Preeclampsia is clinically diagnosed when patients present with new-onset hypertension and one, or more, of the following symptoms: renal involvement (including significant proteinuria (urine protein/creatinine ≥ 30mg/mmol)), haematological, liver or neurological involvement, pulmonary oedema and/or fetal growth restriction (FGR) [7]. Created with BioRender.com

Hypertensive disorders of pregnancy are the leading cause of maternal morbidity and mortality in developing countries [5]. One of the most severe forms of pregnancy-induced hypertension is preeclampsia, which can progress to eclampsia and result in maternal and/or fetal death [5]. Preeclampsia is classified as a disorder of widespread vascular endothelial dysfunction and vasospasm that occurs after 20 weeks’ gestation, with symptoms that can persist until 4–6 weeks post-partum [6]. Preeclampsia is clinically diagnosed when pregnant patients present with new-onset hypertension in conjunction with other symptoms [7] (Fig. 1).

Dysregulation of maternal circulating, intrarenal and placental RASs have been well described in cases of preeclampsia [4, 8, 9]. In particular, abnormal intrauterine RAS activity could contribute to the altered presence and activity of RAS proteins/peptides in the maternal circulation [10, 11]. One RAS component that is less well described but is emerging in interest is the prorenin receptor ((P)RR), a functional element of RAS signalling in tissues. The (P)RR is categorised as a receptor for both renin, the rate-limiting enzyme of the RAS cascade, and its inactive precursor, prorenin [12]. Binding of prorenin to the (P)RR non-proteolytically activates prorenin, enhances the activity of renin, and ultimately leads to increased formation of angiotensin II (Ang II) [12]. The (P)RR can also internalise prorenin and angiotensinogen (AGT), leading to intracellular angiotensin generation [13, 14]. Maternal tissues (apart from the kidney) are incapable of secreting active renin but can produce prorenin [15]. However since only prorenin is produced by tissues, local tissue RASs depend on activation of prorenin, or internalisation of prorenin/(P)RR to initiate RAS signalling [13, 15]. However, the role of (P)RR in activating these pathways in the context of preeclampsia is yet to be explored.

As well as its role in activating RAS in tissues, the extracellular domain of the (P)RR can be cleaved and released into the extracellular space becoming s(P)RR [16], which can also bind renin/prorenin and activate the RAS [16]. Recently, the s(P)RR has been shown to act as an agonist at the AT_1_R [17] and thus can activate the RAS through two pathways. Both placental (P)RR and maternal circulating s(P)RR are elevated in preeclamptic pregnancies [18] and could disrupt the regulation of the intrauterine RAS, maternal iRAS, and circulating RAAS, ultimately interfering with the normal progression of pregnancy. We postulate that high levels of maternal circulating s(P)RR may lead to secondary activation of the maternal RAAS and iRAS, thus contributing to the pathogenesis of preeclampsia.

## The (Pro)renin Receptor and the Soluble (Pro)renin Receptor

### The (Pro)renin Receptor ((P)RR)

The (P)RR (also known as *ATP6AP2*) is a functional receptor of the type 1 transmembrane receptor family, consisting of a large N-terminal extracellular domain, a single transmembrane protein, and a short cytoplasmic domain [19]. The *ATP6AP2* gene is expressed throughout the human brain, placenta, and heart [12], with lower expression within the pancreas, kidney, liver, lung, and skeletal muscle [12].

**Fig. 2:**
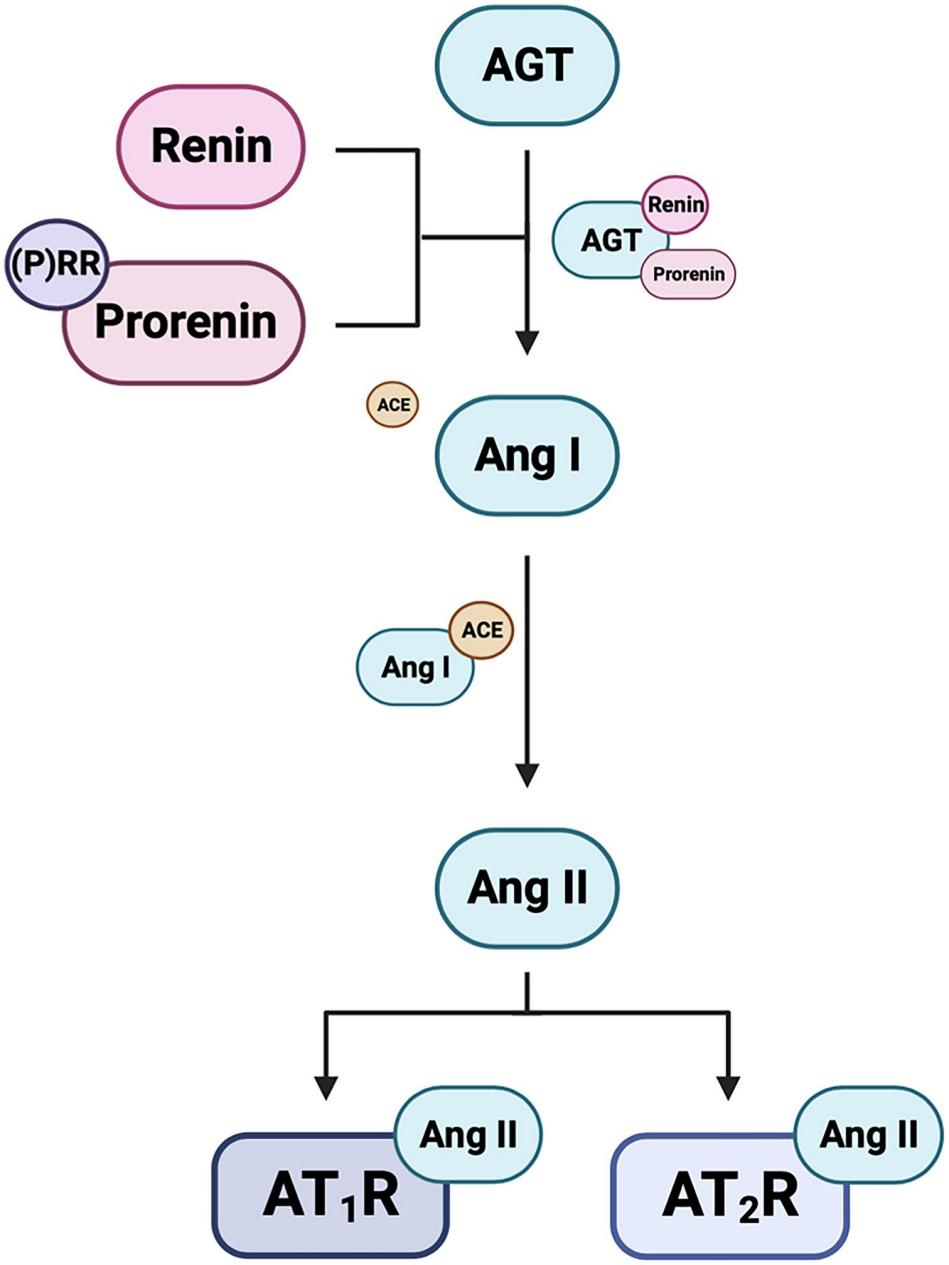
Renin-angiotensin system (RAS) signalling. Renin, or prorenin activated by binding to the (pro)renin receptor ((P)RR), can cleave angiotensin I (Ang I) from angiotensinogen (AGT). Ang I is then converted to Ang II by angiotensin-converting enzyme (ACE). Ang II can either directly activate the Ang II type 1 (AT_1_R) or type II (AT_2_R) receptor. Created with BioRender.com

The (P)RR can bind to both prorenin and renin [20]. For prorenin, this elicits a non-proteolytic conformational change, allowing it to have enzymatic activity [12]. Thus, when prorenin or renin bind to the (P)RR, their enzymatic activity is enhanced, resulting in the cleavage of angiotensin I (Ang I) from AGT [21–23]. Ang I is then converted to angiotensin II (Ang II) by angiotensin-converting enzyme (ACE). The Ang II peptide can bind directly to one of two main receptors, the Angiotensin II type 1 receptor (AT_1_R) or the AT_2_R (Fig. 2) [23]. Ang II/AT_1_R signalling stimulates an inflammatory phenotype promoting vasoconstriction and elevated blood pressure. The effects of Ang II/AT_2_R signalling are physiologically opposite from Ang II/AT_1_R signalling as it stimulates an anti-inflammatory phenotype resulting in vasodilation and decreasing blood pressure [23, 24]. It is important to note that Sun et al*.,* have shown within proximal tubule epithelial cells in vitro, that the endocytic receptor Megalin can internalise renin, prorenin, (P)RR and AGT [13, 14]. Moreover, Tojo et al*.,* highlighted in the podocytes, proximal tubules, and distal nephron, prorenin bound to both (P)RR and megalin were endocytosed in a rat model of diabetes [25]. Together, these studies demonstrate that prorenin can be internalised and activated intracellularly within the lysosome, potentially resulting in intracellular angiotensin generation [13, 14, 25]. Thus, the (P)RR has the potential to activate prorenin both extracellularly and intracellularly.

Besides being involved in RAS signalling, the (P)RR plays multifaceted roles in several essential cellular functions independent of the classical RAS pathway. In vivo studies have shown that prorenin/renin binding to the (P)RR induces ERK1/2 phosphorylation [26], which promotes proliferation, differentiation, apoptosis, and embryogenesis [27]. The transmembrane and cytoplasmic domain of the (P)RR (together known as the M8.9 segment) form an integral part of the vacuolar-type H^+^ adenosine triphosphatase (V-ATPase) complex [28]. The V-ATPase complex helps regulate the cellular microenvironment by maintaining cellular pH [29]. Additionally, the (P)RR facilitates the interaction between the V-ATPase complex and Wnt receptors, frizzled 8 (FZD8) and lipoprotein receptor-related protein 6 (LRP6) [30]. Acidification mediated by V-ATPase is essential for LRP6 phosphorylation and subsequent activation of β-catenin signalling [30]. This is necessary for proper placentation during early pregnancy, by stimulating trophoblast proliferation, migration, and invasion [31]. Furthermore, the extracellular domain of full-length (P)RR is also able to bind pyruvate dehydrogenase (PDH) via the PDHB subunit, and prevents PDH degradation while supporting its activity [32]. Interestingly, in vitro knockdown of *ATP6AP2* gene expression in human retinal epithelial cells results in a reduction in PDH activity, increased lactate levels and impaired glucose-stimulated oxidative stress [32]. This suggests that the (P)RR plays a role in aerobic glucose metabolism, whether this is influenced by renin/prorenin binding is yet to be determined. Although these RAS independent actions of the (P)RR may be important in fetal and placental development and function, this review only focuses on the role(s) of (P)RR that are related to the placental, circulating, and/or intrarenal RASs during pregnancy and within the pathology of preeclampsia.

### The Soluble (Pro)renin Receptor (s(P)RR)

Full length (P)RR can be cleaved and secreted as a soluble form, s(P)RR. Proteases including furin [16, 33], ADAM 19 [34], and site 1 protease (MBPTS1) [33, 35], mediate the cleavage of the extracellular domain from the transmembrane domain of full length (P)RR in the Golgi [16]. The newly released 28 kDa extracellular domain, s(P)RR, has been detected in both the plasma and urine [16, 36]. While the functions of s(P)RR are largely uncharacterised, recent studies have demonstrated that s(P)RR is functional. Like full-length (P)RR, the s(P)RR is able to bind circulating renin and prorenin and subsequently activate the circulating and possibly tissue RASs [37].

Evidence suggests that s(P)RR acts as a paracrine factor [17, 38]. Urinary s(P)RR is elevated in rats undergoing water deprivation and is associated with enhanced membrane bound (P)RR in the cortex and inner medulla of the rat kidney [39]. Furthermore, in vitro treatment of primary rat inner medullary collecting duct cells with recombinant s(P)RR stimulated aquaporin 2 mRNA expression [36]. As such, s(P)RR can act in a paracrine fashion to regulate fluid retention and pH balance [40] within the collecting duct. Treatment with recombinant s(P)RR also increases systolic blood pressure in high fat-fed male mice [17, 41]. This increase in blood pressure was postulated to be mediated through the actions of s(P)RR on baroreflex sensitivity, enhancing sympathetic nerve activity through the release of leptin. Furthermore, in a novel mouse model using CRISPR-Cas9 to mutate the cleavage site of the (P)RR to reduce circulating s(P)RR levels [42, 43], mutant mice treated with aldosterone-salt became largely resistant to hypertension and plasma volume expansion [43]. Additionally, Ang II-induced hypertension and renal injury was blunted in mutant mice that lacked s(P)RR [42, 44]. Recombinant s(P)RR has been shown to increase epithelial sodium channel (ENaC) activity in collecting duct cells in vitro [45], while nephron-specific deletion of intact (P)RR in vivo resulted in increased urinary sodium excretion with reduced ENaC abundance and activity [46]. As such, s(P)RR may mediate Ang II-induced hypertension through enhancing intrarenal ENaCs [44, 45], demonstrating that s(P)RR plays a crucial role in salt/water balance, blood pressure regulation, and renal function.

**Fig. 3:**
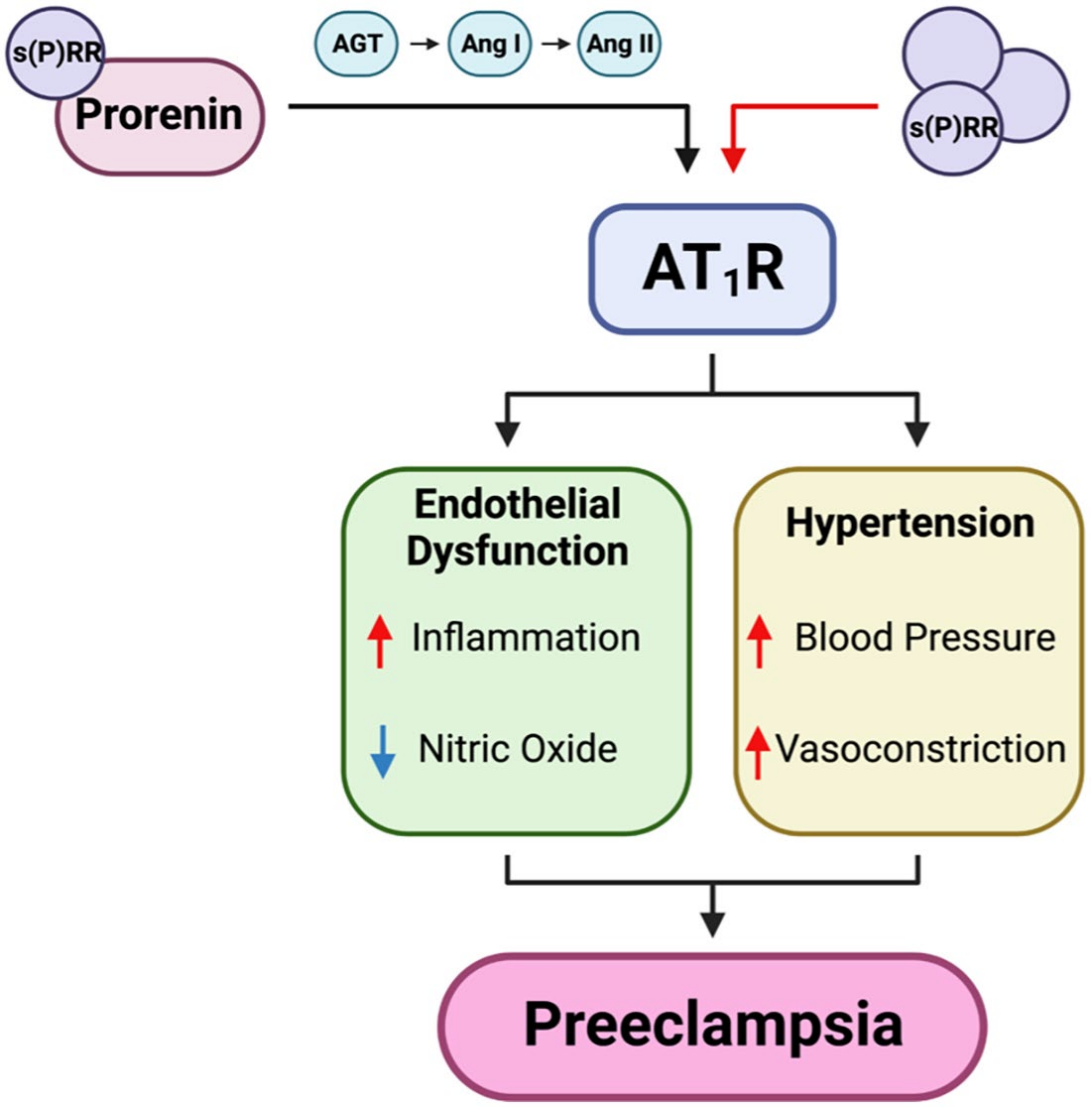
The agonistic effects of soluble (pro)renin receptor (s(P)RR) on the renin-angiotensin system (RAS). The soluble prorenin receptor (s(P)RR), can bind prorenin and enhance its enzymatic activity through non-proteolytic conformational change. Prorenin can then cleave angiotensin I (Ang I) from angiotensinogen (AGT) and initiate RAS signalling. The s(P)RR can also act as a direct agonist for the Ang II type 1 receptor (AT_1_R). AT_1_R activation results in hypertension and endothelial dysfunction, the two main symptoms of preeclampsia. Red arrows indicate increased activity/expression and blue arrows represent decreased activity/expression. Created with BioRender.com

Unlike full-length membrane bound (P)RR, the s(P)RR has been demonstrated to be a direct agonist for the AT_1_R [17], disrupting the notion that Ang peptides are the sole activating ligands for the dominant receptor of the RAS cascade [23, 47]. Overstimulation of AT_1_R is implicated as a contributing factor for the development of cardiovascular disease, including hypertension [48]. Interestingly, Fu et al., showed that s(P)RR directly binds to AT_1_R, suppressing nitric oxide (NO) generation in endothelial cells [17]. Under normal physiological conditions, NO has an anti-inflammatory effect [49]. These findings suggest that elevated s(P)RR might cause inflammation in the vascular endothelium, which is a feature of preeclampsia (Fig. 3) [17]. However, further investigation is required to confirm that s(P)RR does act as a direct AT_1_R agonist and better understand the role s(P)RR/AT_1_R signalling plays in the regulation of blood pressure and endothelial dysfunction during pregnancy.

## The (Pro)renin Receptor and the Renin-angiotensin System in Pregnancy

### Placental Renin-Angiotensin System and the (Pro)renin Receptor

#### Normotensive Pregnancy

Placental RAS signalling is essential for normal placentation. As the placenta develops, human chorionic gonadotrophin (hCG) secretion increases, stimulating ovarian [50] and placental prorenin production [51], which could facilitate early placental RAS signalling. Placental RAS expression is variable throughout pregnancy with the highest expression of AGT, renin, (P)RR, and AT_1_R, mRNAs and Ang II peptides evident in the first trimester, with a subsequent decrease towards term [52, 53]. In contrast, placental ACE mRNA expression, which is only expressed on the fetal endothelium, is highest at term [52]. The components of the RAS are also differentially expressed within placental cells of the chorionic villi. Both cytotrophoblasts and syncytiotrophoblasts express most major RAS components (prorenin, (P)RR, AGT, ACE, AT_1_R, AT_2_R) [52, 54–59]. However, syncytiotrophoblasts do not express the AT_2_R, and the AT_1_R has only been measured at low levels [52, 55]. Furthermore, AT_1_R and AT_2_R are not expressed in cytotrophoblasts at either the mRNA or protein level [54, 55, 59]. Notably, soluble forms of (P)RR, and ACE are secreted by cytotrophoblasts [54, 55, 59, 60], and can enter the maternal circulation and influence the maternal RAS. Extravillous trophoblasts have been shown to express all Ang II receptors [52, 55, 58] and produce (P)RR and prorenin at the protein level [31, 51] and thus are likely to be the major site of local RAS actions.

The nature of (P)RR in relation to both placental and fetal development has been well documented. During early fetal development, (P)RR is considered essential for fetal kidney development, with a complete reduction of (P)RR being embryonically lethal [61, 62]. In regards to the placenta, evidence from our research group has demonstrated, in a first trimester extravillous trophoblast cell line (HTR-8/SVneo) model, that knocking down (P)RR gene expression decreased trophoblast invasion and migration in vitro [63]. These data were supported by further studies by our research group in pregnant mice, where lentiviral knockdown of (P)RR within the placenta reduced total trophoblast cell number and decreased syncytiotrophoblast thickness [63]. Mice with placental-specific (P)RR deficiency also had reduced placental functional capacity and fetal viability [63], highlighting that trophoblast (P)RR expression is key for healthy placental development.

Ang II/AT_1_R signalling is crucial for placental development. First-trimester placental explants show an increase in extravillous cytotrophoblast proliferation in vitro in response to Ang II treatment [64, 65], and this effect was abolished following pharmacological blockade of the AT_1_R, with Olmesartan [65]. Additionally, Ang II signalling promotes differentiation of decidual cells, increasing endometrial cell permeability in vivo [66], and allowing for trophoblast invasion into the maternal endometrium. Previous studies using AT_1_R deficient rodents, demonstrated that AT_1_R is necessary for appropriate trophoblast development, angiogenesis and, subsequently, placental function [67]. Together, these studies implicate Ang II/AT_1_R signalling in trophoblast proliferation and invasion into the maternal endometrium to facilitate implantation [12, 52].

#### Preeclamptic Pregnancy

Preeclamptic pregnancies are characterised by shallow placentation coupled with reduced uteroplacental perfusion leading to placental hypoxia/reperfusion events [68, 69]. These fluctuations in oxygen tension cause an increase in the production of reactive oxygen species (ROS) [70] as well as a variety of anti-angiogenic factors that are released into the maternal circulation. Both placental (P)RR protein levels and maternal plasma s(P)RR levels are elevated at term in preeclamptic pregnancies compared with normotensive pregnancies [18]. These findings implicate (P)RR and s(P)RR in the pathogenesis of preeclampsia, however no correlation was observed between placental (P)RR expression and plasma s(P)RR levels in patients with preeclampsia [18]. As such, increases in both placental (P)RR and plasma s(P)RR may be independent of each other. Interestingly, within placental biopsies from preeclamptic pregnancies, both elevated (P)RR levels and increased oxidative stress have been shown to enhance the cleavage of Ang I from AGT [71, 72]. These studies suggest that elevated placental (P)RR levels, as seen in the preeclamptic placenta, may enhance local uteroplacental RAS signalling.

The functional changes in uteroplacental RAS signalling in preeclampsia remains to be fully understood. Studies by Mistry et al*.,* and Herse et al*.,* highlighted that the only functional change in RAS signalling between preeclamptic and normotensive placenta was elevated AT_1_R protein levels [71, 73]. In contrast, Shah et al*.,* have shown an increase in renin expression in the decidua of preeclamptic patients [74], suggesting that the maternal decidua may act as an additional site of uteroplacental RAS activation. Moreover, term chorionic villous explants from preeclamptic pregnancies exhibit elevated Ang II levels when compared with normotensive pregnancies in vitro [53]. Together, these studies show an increased activation of RAS signalling but highlight the need for further studies in the context of preeclampsia.

### Circulating Renin-Angiotensin System and the (Pro)renin Receptor

#### Normotensive Pregnancy

As pregnancy progresses, maternal physiological adaptations occur in response to the growing metabolic demands of the fetus and placenta, leading to a greatly expanded cardiovascular system [75]. As such, during early pregnancy there is a decrease in maternal blood pressure coupled with an increase in renal blood flow and glomerular filtration rate [75]. During early gestation, increased circulating prorenin [76], renin [77, 78], AGT [1], ACE [54], Ang I [77], Ang II [77], and aldosterone [77, 79], are essential for maintaining fluid and electrolyte homeostasis and blood pressure throughout gestation. Prorenin produced by the ovary is the most notable increase during early gestation [76]. However, given that prorenin is inactive, elevated Ang II levels are likely increased as a consequence of liver secreted AGT in response to elevated estrogen [1]. Interestingly, elevated prorenin may trigger a decrease in (P)RR levels, as prorenin has been shown to control (P)RR expression via a negative feedback mechanism [80, 81]. Despite this early increase in RAS components, there is a reduction in its vasoconstrictor and vasopressor actions. This is the result of down-regulation of vascular AT_1_R, which decreases the vasoconstrictor actions of Ang II/AT_1_R signalling, and dampens vascular reactivity to Ang II [82, 83]. Alternatively, both Ang II/AT_2_R and Ang-(1–7)/Mas receptor signalling promote vasodilation throughout gestation [77, 84]. Collectively, the altered vascular environment helps maintain blood pressure/cardiac output to sustain uteroplacental perfusion throughout pregnancy.

Within the maternal circulation, plasma s(P)RR concentrations progressively increase from the first trimester until term [85]. Furthermore, increases in plasma s(P)RR concentrations in early pregnancy can predict systolic/diastolic blood pressure elevation in late gestation [85]. However, plasma s(P)RR concentrations from middle to late pregnancy are not associated with changes in blood pressure [85]. Conversely, Nartita et al., showed that early plasma s(P)RR concentrations alone were not sufficient to predict elevated systolic blood pressure at term [18], but placental (P)RR expression and plasma s(P)RR levels combined were [18]. Hence, the nature of circulating plasma s(P)RR concentrations and maternal blood pressure in pregnancy remains to be fully understood.

#### Preeclamptic Pregnancy

The s(P)RR has become of interest in preeclamptic pregnancies as it is elevated in the maternal plasma of preeclamptic patients [18, 85, 86]. This has been disputed however by Sugulle et al*.,* who reported that s(P)RR is dysregulated in pregnancies affected by diabetes mellitus, but not preeclampsia [87]. This could be due to differences in ethnicity or preeclampsia diagnostic criteria between cohorts. Nonetheless, other studies have reported that s(P)RR levels in the maternal plasma are higher in early gestation in patients with preeclampsia compared with normotensive pregnancies and continue to increase until term [85, 88], indicating its potential role in the pathology of preeclampsia.

The role of the elevated s(P)RR in preeclamptic pregnancies is relatively unstudied. Research by Nartita et al*.,* indicates that s(P)RR may decrease renal function as elevated plasma s(P)RR levels in preeclamptic patients are negatively correlated with estimated glomerular filtration rate (eGFR) [18]. Furthermore, evidence from our laboratory has illustrated that human uterine microvascular endothelial cells (HUtMECs) treated with recombinant human s(P)RR exhibited increased expression of endothelial dysfunction markers (vascular cellular adhesion molecule-1, intracellular adhesion molecule-1, and endothelin-1) and impaired vascular formation [89]. Treatment of HUtMECs with recombinant human s(P)RR also increased adhesion of human peripheral blood mononuclear cells to endothelial cells in vitro [89]. As such, we postulate that elevated s(P)RR produces endothelial dysfunction, promoting vascular injury. A recent study by Fu etal., has shown that s(P)RR induced endothelial dysfunction through s(P)RR binding with the AT_1_R in vitro [17]. Interestingly Fu et al., highlighted, in a non-pregnant high-fat fed mouse model, that s(P)RR treatment increased mean arterial blood pressure, systolic blood pressure, and diastolic blood pressure [17]. Moreover, endothelium-dependent vasorelaxation in mesenteric arteries in response to acetylcholine was significantly diminished in the s(P)RR treated group [17]. Ramkumar et al., highlighted that in s(P)RR deficient mice, mesenteric arteries displayed reduced vasoconstriction following Ang II infusion in conjunction with greater acetylcholine induced vasorelaxation [42]. Collectively, these studies reinforce the connection between s(P)RR and endothelium-dependent regulation of blood pressure. Further studies from our research group have been able to confirm that s(P)RR treatment is associated with elevated maternal blood pressure and decreased fetal growth in pregnant rats [89]. As such, there may be a concentration specific relationship between circulating maternal s(P)RR levels and fetal development, however the exact mechanisms remain to be fully understood. Additionally, we have shown that isolated maternal renal arteries displayed a decreased sensitivity to acetylcholine induced vasodilation [89]. These studies demonstrate the potential role elevated s(P)RR, via activation of the RAS could play in the pathogenesis of hypertension, vascular dysfunction and more specifically, the poor outcomes seen in preeclamptic pregnancies.

Maternal circulating RAS components are also altered in preeclamptic pregnancies [4, 90]. Preeclamptic patients have elevated circulating prorenin levels throughout gestation [1, 85], which may underpin the activation of the circulating RAS or be taken up intracellularly to activate tissue RAS. The (P)RR can enhance the catalytic activity of renin/prorenin, promoting the formation of Ang I from AGT [91]. AGT can also exist in an oxidised state, which has a higher affinity for renin than reduced AGT [91] and in this instance the presence of the (P)RR/s(P)RR further enhances AGT’s affinity for renin [1]. Elevated s(P)RR levels in the plasma of preeclamptic patients can increase the activity of the circulating RAS and iRAS. Notably, preeclamptic pregnancies are also reported to have reduced circulating levels of renin, ACE, Ang I, and Ang II compared with normotensive controls [90]. Because of this, patients with preeclampsia develop a heightened sensitivity to Ang II/AT_1_R signalling within the first 10 weeks of pregnancy [83]. Enhanced Ang II sensitivity is suggested to be due to heterodimerisation of the AT_1_R with the bradykinin receptor [92, 93], which has been shown to be resistant to inactivation by reactive oxygen species while also being hyper responsive to Ang II [92, 94]. Additionally, AT_1_R signalling is increased, as autoantibodies for AT_1_R are significantly elevated in preeclampsia [95–97] and have been shown to have the same actions as Ang II (i.e., can bind to and activate the AT_1_R) [98]. Furthermore, as described above, s(P)RR, which is elevated in preeclamptic pregnancies, has been shown to bind to the AT_1_R and promote its signalling [17]. Enhanced Ang II sensitivity in conjunction with the presence of AT_1_R autoantibodies and s(P)RR/AT_1_R signalling could substantially increase AT_1_R signalling and thus activate the maternal circulating RAAS and influence maternal blood pressure in preeclamptic pregnancies. It is important to note however, no studies have explored the interactions between s(P)RR and AT_1_R autoantibodies and it is likely they both compete for binding to the AT_1_R.

### Intrarenal Renin-Angiotensin System and the (Pro)renin Receptor

#### Normotensive Pregnancy

Intrarenal RAS activation undergoes specific and necessary changes to sustain a healthy pregnancy. Active renin is released from juxtaglomerular cells within the kidney into the maternal circulation [99]. Cellular release of active renin is dependent upon; renal baroreceptor stimulation, the sympathetic nervous system, and the sodium levels circulating within the distal tubule [99]. Collectively, this region is known as the juxtaglomerular apparatus (JGA). Low sodium within the JGA stimulates renin release [100], leading the iRAS to play a regulatory role in controlling the activity of the maternal circulating RAAS, and maintain fluid/electrolyte and cardiovascular homeostasis [1]. As such, throughout gestation, active circulating renin levels increase as demands for Ang II and aldosterone increase to help maintain circulating blood volume during pregnancy [90].

In conjunction with JGA-mediated renin release, the iRAS regulates sodium homeostasis and blood pressure by influencing renal tubular sodium reabsorption. The iRAS functions primarily through Ang II/AT_1_R signalling to mediate increased Ang II uptake within the proximal tubule. Additionally, active renin or prorenin acting on the (P)RR, can lead to an increase in Ang II production and enhanced RAS signalling [22]. The increased Ang II levels stimulate the production and uptake of AGT in the proximal tubule, resulting in increased production of Ang II in the distal segments of the nephron [101]. Ang II activation of AT_1_R increases distal Na^+^ reabsorption in the kidneys. As well, circulating Ang II stimulates aldosterone release from the adrenal cortex [102]. Ang II/AT_1_R-mediated signalling increases vasopressin release from the posterior pituitary gland, which promotes reabsorption of water by the collecting duct, salt appetite and thirst; collectively leading to an increase in total blood volume [103–105]. Interestingly, a rat model of pregnancy displayed a renal cortical and inner medullary increase in (P)RR protein levels in conjunction with elevated urinary s(P)RR towards term gestation [106]. This suggests that (P)RR levels within the kidney could be reflective of kidney health, in relation to urinary protein concentrations.

#### Preeclamptic Pregnancy

For iRAS activation, Ang II signalling is key. In vivo rat studies have shown that tissue specific elevations in intrarenal Ang II levels resulted from AT_1_R mediated Ang II uptake in proximal tubules, stimulating the iRAS to increase intratubular production of Ang II, increasing distal Na^+^ reabsorption and causing renal damage [107, 108]. The s(P)RR can bind and activate the AT_1_R [17], hence elevated serum s(P)RR levels observed in preeclamptic pregnancies may result in increased AT_1_R stimulation [17], similarly affecting the iRAS. Additionally, elevated maternal plasma s(P)RR levels in preeclamptic patients are negatively correlated with estimated glomerular filtration rate [18], highlighting an association between elevated s(P)RR and renal dysfunction, a key symptom of preeclampsia. In a CRISPR-Cas9 mouse model mutating the cleavage site of the (P)RR such that s(P)RR is not generated, the loss of s(P)RR attenuated Ang II induced hypertension while also reducing albuminuria and renal tubular injury [42]. Thus highlighting that reduced s(P)RR may be protective against Ang II-induced renal injury. Whether urinary s(P)RR is increased in preeclamptic or normotensive pregnancy remains to be seen [86]. In a mouse of model of 5/6 nephrectomy (an experimental subtotal nephron ablation model of induced chronic kidney disease), mice displayed an increase in urinary/renal levels of renin, AGT, and Ang II [109]. In the same model, treating mice with a (P)RR antagonist PRO20, reduced urinary/renal protein levels of renin, AGT, and Ang II while impairing active-β-catenin within the renal cortex. These outcomes suggest the (P)RR mediates renal injuries through iRAS activation and/or β-catenin signalling [109], highlighting a potential role of the (P)RR in renal injury seen in preeclamptic pregnancies.

## Management and Therapeutic Strategies for Preeclampsia

### (Pro)renin Receptor/Soluble (Pro)renin Receptor as a Biomarker for the Early Detection of Preeclampsia

In recent years, preeclampsia screening has focused on circulating biomarkers of maternal and/or placental origin [110, 111]. Elevated levels of anti-angiogenic factors have proven to be useful in predicting early onset preeclampsia (sFLT1, sENG, and PlGF, ratios), allowing clinicians to predict the severity of the pathology in addition to identifying the need for early delivery [112]. However, the predictability of these ratios is dependent upon when they are measured during gestation. Patients pre-destined to develop preeclampsia exhibit no significant increase in sFLT1/PIGF and sENG/PIGF ratios until 20 weeks of gestation [113]. This leaves a crucial period during early placental development, without any predictive biomarkers to aid in determining the health of the pregnancy. As such, potential novel therapeutic biomarkers such as (P)RR/s(P)RR, could provide early and more robust knowledge that could inform clinical care and management.

Elevated placental (P)RR protein levels are associated with increased systolic blood pressure at term [18]. Additionally, high plasma s(P)RR levels in early pregnancy (< 16 weeks) have been shown to predict higher systolic blood pressures in mid-late pregnancy [41, 85]. Thus, increased plasma s(P)RR in the first trimester could be a useful biomarker to predict the onset of hypertension in preeclampsia. However, further investigation is required to understand the relationship between blood pressure and plasma s(P)RR levels and much larger studies are required to determine if s(P)RR levels in the first trimester can predict preeclampsia.

### (Pro)renin Receptor/Soluble (Pro)renin Receptor as a Potential Therapeutic for Preeclampsia

The only effective treatment for preeclampsia is the removal of the placenta and delivery of the baby [114]. Consequently, preeclamptic pregnancies may result in early deliveries, which predispose preterm infants to a greater likelihood of poor long-term health outcomes. Currently, low dose aspirin treatment is effective in secondary prevention of preeclampsia in patients with a history of preeclampsia [115], with remaining therapeutic options focusing on symptom management.

Symptoms of overt hypertension can be attenuated in pregnancy using anti-hypertensive medications including: Nifedipine (calcium channel blocker) [116], Labetalol (beta blocker) [117] and Methyldopa (alpha blocker) [118]. Investigation into which form of medication is the most effective for patients suffering from severe early and late onset preeclampsia is still ongoing and a consensus has not yet been determined [119]. Additionally, using loading doses of magnesium sulphate is proven to be an effective and safe therapeutic and anticonvulsant in preeclampsia and eclampsia [120]. Collectively, both antihypertensive and anticonvulsant treatments for preeclampsia focus on reducing the symptoms of preeclampsia however, these treatments are only functionally effective for patients presenting with severe onset preeclampsia and/or patients with a history of the pathology [121].

Treatments targeting RAS signalling in preeclampsia, and more specifically (P)RR, need to be considered carefully as RAS antagonists can cross the placenta and affect key RAS signalling pathways in the developing fetus (particularly renal development) [122]. Therefore, traditional antihypertensives that target the RAS (ACE inhibitors etc.) are contraindicated in pregnancy [122–124]. Targeting the high levels of placental (P)RR and/or quenching excess circulating s(P)RR specifically could provide an alternative to traditional anti-hypertensive drugs in the treatment of preeclampsia. Adopting the use of PEG-PLA nanoparticle drug delivery systems, for example, could provide a novel siRNA delivery system to manipulate placental-specific gene expression [125]. This could reduce placentally derived s(P)RR and subsequently reduce vascular and intrarenal s(P)RR/AT_1_R signalling [17], reducing the hypertensive symptoms seen in preeclampsia. As previously stated, mutant mice that lack s(P)RR are resistant to aldosterone-salt or Ang II-induced hypertension and renal injury [42–44]. Thus, targeting proteases required for s(P)RR cleavage (such as site 1 protease inhibitor, PF429242), could provide a novel therapeutic option to quench excess circulating s(P)RR [126] and mediate hypertensive symptoms seen in preeclamptic pregnancies. Studies from Morosin et al*.,* showed that treatment of primary human placental trophoblasts with the protease inhibitor DEC-RVKR-CMK (which inhibits the activity of pro-protein convertase subtilisin/kexin’s (PCSK) 1–7, including furin) significantly reduces extracellular s(P)RR protein secretion [60, 127, 128]. Interestingly, this effect was not observed with a specific siRNA knockdown of FURIN expression or MBTPS1 (site 1 protease) inhibition [60]. Additionally, post DEC-RVKR-CMK treatment, both (P)RR and intracellular s(P)RR protein expression remained unchanged. This suggests that this method of protease inhibition may only inhibit proteases responsible for the final maturation of s(P)RR prior to secretion [60] and not the initial cleavage. As such, protease inhibition could decrease placental s(P)RR secretion, leading to reduced iRAS activation and a reduction of the maternal symptoms of preeclamptic pregnancies. However, further examination of therapeutic strategies specifically targeting the s(P)RR throughout pregnancy are required to be adopted clinically.

Greater focus has been placed on directly targeting the (P)RR in recent years. Antagonistic peptides such as PRO20 and Handle Region decoy Peptide (HRP) can block the binding of renin or prorenin with the (P)RR [129]. Both PRO20 and HRP compete to bind with the handle region of either renin or prorenin, preventing (P)RR binding and subsequently inhibiting s(P)RR/(P)RR signalling [109]. As discussed above, the (P)RR may mediate renal injuries through iRAS activation [109]. As such, preeclamptic pregnancies could see a reduction in maternal symptoms through PRO20 mediated reduction in circulating s(P)RR-induced renin activity or iRAS activity [109, 129]. Mishima et al., highlighted, in a reduced uterine perfusion pressure preeclampsia mouse model, that treatment with the (P)RR antagonist, HRP, suppressed the significant increases in blood pressure and proteinuria while also decreasing markers of endothelial dysfunction [130]. Furthermore, in an elevated sFLT-1 preeclampsia mouse model, (P)RR and Endothelin-1 expression were significantly increased after sFLT-1 infusion, with HRP treatment rescuing these increases [131]. (P)RR decoy peptides (PRO20 and HRP) could prove to be useful therapeutic options for preeclampsia. However, more research is required to understand the functional effects of (P)RR/s(P)RR antagonists during pregnancy.

## Conclusion

In conclusion, this review has examined how the (P)RR/s(P)RR are involved in placental, circulating, and intrarenal RAS throughout pregnancy and demonstrated strong evidence that (P)RR and s(P)RR are involved in the clinical manifestations of preeclampsia. Targeting s(P)RR with an siRNA targeted to the placenta or antagonistic peptides such as PRO20, may reduce plasma s(P)RR in the maternal circulation. Hence s(P)RR inhibition could be an effective therapeutic option for preeclampsia.

## Data Availability

The authors confirm that the data supporting the findings of this study are available within the article.

## References

[1] Lumbers ER, Pringle KG. Roles of the circulating renin-angiotensin-aldosterone system in human pregnancy. Am J Physiol Regul Integr Comp Physiol. 2014;306:R91–101. 24089380 10.1152/ajpregu.00034.2013

[2] Chesley LC. Hypertensive disorders in pregnancy. (Appleton-Century-Crofts, 1978).

[3] Seldin D, Giebisch G. kidney physiology and pathology, vol. 3. New York: Raven Press; 1992.

[4] Irani RA, Xia Y. Renin angiotensin signaling in normal pregnancy and preeclampsia. in Seminars in nephrology, Vol. 31 47–58 (Elsevier, 2011). 10.1016/j.semnephrol.2010.10.005 PMC3275085 21266264

[5] Duley L. The global impact of pre-eclampsia and eclampsia. in Seminars in perinatology, Vol. 33 130–137 (Elsevier, 2009). 10.1053/j.semperi.2009.02.010 19464502

[6] Young BC, Levine RJ, Karumanchi SA. Pathogenesis of preeclampsia. Annu Rev Pathol. 2010;5:173–92. 20078220 10.1146/annurev-pathol-121808-102149

[7] Lowe SA, et al. SOMANZ guidelines for the management of hypertensive disorders of pregnancy 2014. Aust N Z J Obstet Gynaecol. 2015;55:e1–29. 26412014 10.1111/ajo.12399

[8] Irani RA, Xia Y. The functional role of the renin–angiotensin system in pregnancy and preeclampsia. Placenta. 2008;29:763–71. 18687466 10.1016/j.placenta.2008.06.011 PMC2614866

[9] Pringle KG, et al. Urinary angiotensinogen excretion in Australian Indigenous and non-Indigenous pregnant women. Pregnancy Hypertension. 2018;12:110–7. 29674190 10.1016/j.preghy.2018.04.009

[10] Herse F, et al. Dysregulation of the circulating and tissue-based renin-angiotensin system in preeclampsia. Hypertension. 2007;49:604–11. 17261642 10.1161/01.HYP.0000257797.49289.71

[11] Pringle KG, Lumbers ER, Morosin SK, Delforce SJ. The role of angiotensins in the pathophysiology of human pregnancy. in *Angiotensin* 179–211 (Elsevier, 2023).

[12] Nguyen G, et al. Pivotal role of the renin/prorenin receptor in angiotensin II production and cellular responses to renin. J Clin Investig. 2002;109:1417–27. 12045255 10.1172/JCI14276 PMC150992

[13] Sun Y, et al. Megalin: a novel endocytic receptor for prorenin and renin. Hypertension. 2020;75:1242–50. 32200675 10.1161/HYPERTENSIONAHA.120.14845

[14] Sun Y, Lu X, Danser AJ. Megalin: a novel determinant of renin-angiotensin system activity in the kidney? Curr Hypertens Rep. 2020;22:1–7. 32172431 10.1007/s11906-020-01037-1 PMC7072043

[15] Nehme A, Zouein FA, DerisZayeri Z, Zibara K. An update on the tissue renin angiotensin system and its role in physiology and pathology. J Cardiovasc Dev Dis. 2019;6:14. 30934934 10.3390/jcdd6020014 PMC6617132

[16] Cousin C, et al. Soluble form of the (pro) renin receptor generated by intracellular cleavage by furin is secreted in plasma. Hypertension. 2009;53:1077–82. 19380613 10.1161/HYPERTENSIONAHA.108.127258

[17] Fu Z, et al. Soluble (pro) renin receptor induces endothelial dysfunction and hypertension in mice with diet-induced obesity via activation of angiotensin II type 1 receptor. Clin Sci. 2021;135:793–810. 10.1042/CS20201047 PMC9215112 33625485

[18] Nartita T, et al. Placental (pro) renin receptor expression and plasma soluble (pro) renin receptor levels in preeclampsia. Placenta. 2016;37:72–8. 26684753 10.1016/j.placenta.2015.11.007

[19] Burcklé C, Bader M. Prorenin and its ancient receptor. (Am Heart Assoc. 2006). 10.1161/01.HYP.0000241132.48495.df 16940209

[20] Nguyen G, et al. Pivotal role of the renin/prorenin receptor in angiotensin II production and cellular responses to renin. J Clin Invest. 2002;109:1417–27. 12045255 10.1172/JCI14276 PMC150992

[21] Nguyen G. Renin/prorenin receptors. Kidney Int. 2006;69:1503–6. 16672920 10.1038/sj.ki.5000265

[22] Oshima Y, Morimoto S, Ichihara A. Roles of the (pro)renin receptor in the kidney. World J Nephrol. 2014;3:302–7. 25374826 10.5527/wjn.v3.i4.302 PMC4220365

[23] Chappell MC. Biochemical evaluation of the renin-angiotensin system: the good, bad, and absolute? Am J Physiol Heart Circ Physiol. 2016;310:H137–52. 26475588 10.1152/ajpheart.00618.2015 PMC4796631

[24] Tamanna S, Lumbers ER, Morosin SK, Delforce SJ, Pringle KG. ACE2: a key modulator of the renin-angiotensin system and pregnancy. Am J Physiol Regul Integr Comp Physiol. 2021;321:R833–43. 34668428 10.1152/ajpregu.00211.2021 PMC8862784

[25] Tojo A, Kinugasa S, Fujita T, Wilcox CS. A local renal renin–angiotensin system activation via renal uptake of prorenin and angiotensinogen in diabetic rats. Diabetes Metab Syndr Obes Targets Ther. 2016;1–10. 10.2147/DMSO.S91245 PMC4723098 26848273

[26] Reyes-Martinez C, Nguyen QM, Kassan M, Gonzalez AA. (Pro)renin Receptor-Dependent Induction of Profibrotic Factors Is Mediated by COX-2/EP4/NOX-4/Smad Pathway in Collecting Duct Cells. Front Pharmacol. 2019;10:803. 31396082 10.3389/fphar.2019.00803 PMC6664006

[27] Mebratu Y, Tesfaigzi Y. How ERK1/2 activation controls cell proliferation and cell death: Is subcellular localization the answer? Cell Cycle. 2009;8:1168–75. 19282669 10.4161/cc.8.8.8147 PMC2728430

[28] Peters J. The (pro) renin receptor and its interaction partners. Pflüg Arch Eur J Physiol. 2017;469:1245–56. 10.1007/s00424-017-2005-z 28620832

[29] Pamarthy S, Kulshrestha A, Katara GK, Beaman KD. The curious case of vacuolar ATPase: regulation of signaling pathways. Mol Cancer. 2018;17:1–9. 29448933 10.1186/s12943-018-0811-3 PMC5815226

[30] Ichihara A, Yatabe MS. The (pro) renin receptor in health and disease. Nat Rev Nephrol. 2019;15:693–712. 31164719 10.1038/s41581-019-0160-5

[31] Pollheimer J, et al. Activation of the canonical wingless/T-cell factor signaling pathway promotes invasive differentiation of human trophoblast. Am J Pathol. 2006;168:1134–47. 16565489 10.2353/ajpath.2006.050686 PMC1606554

[32] Kanda A, Noda K, Ishida S. ATP6AP2/(pro) renin receptor contributes to glucose metabolism via stabilizing the pyruvate dehydrogenase E1 β subunit. J Biol Chem. 2015;290:9690–700. 25720494 10.1074/jbc.M114.626713 PMC4392269

[33] Suda C, Yatabe J, Yatabe M, Yarita M, Ichihara A. Soluble (pro) renin receptor increased by hypoxia maintains oxidative metabolism in trophoblasts. J Mol Endocrinol. 2020;64:145–54. 31958319 10.1530/JME-19-0050

[34] Yoshikawa A, et al. The (pro) renin receptor is cleaved by ADAM19 in the Golgi leading to its secretion into extracellular space. Hypertens Res. 2011;34:599–605. 21270819 10.1038/hr.2010.284

[35] Nakagawa T, et al. Site-1 protease is required for the generation of soluble (pro) renin receptor. J Biochem. 2017;161:369–79. 28013223 10.1093/jb/mvw080

[36] Lu X, et al. Soluble (pro) renin receptor via β-catenin enhances urine concentration capability as a target of liver X receptor. Proc Natl Acad Sci. 2016;113:E1898–906. 26984496 10.1073/pnas.1602397113 PMC4822598

[37] Nguyen G, et al. Plasma soluble (pro) renin receptor is independent of plasma renin, prorenin, and aldosterone concentrations but is affected by ethnicity. Hypertension. 2014;63:297–302. 24218434 10.1161/HYPERTENSIONAHA.113.02217

[38] Yang T. Soluble (Pro)Renin Receptor in Hypertension. Nephron. 2023;147:234–43. 35871512 10.1159/000525635 PMC9867785

[39] Wang F, et al. Antidiuretic action of collecting duct (pro) renin receptor downstream of vasopressin and PGE2 receptor EP4. J Am Soc Nephrol. 2016;27:3022–34. 27000064 10.1681/ASN.2015050592 PMC5042659

[40] Sasaki N, Morimoto S, Suda C, Shimizu S, Ichihara A. Urinary soluble (pro) renin receptor excretion is associated with urine pH in humans. PLoS ONE. 2021;16:e0254688. 34310595 10.1371/journal.pone.0254688 PMC8312976

[41] Gatineau E, Gong MC, Yiannikouris F. Soluble prorenin receptor increases blood pressure in high fat–fed male mice. Hypertension. 2019;74:1014–20. 31378099 10.1161/HYPERTENSIONAHA.119.12906 PMC6739191

[42] Ramkumar N, et al. Loss of soluble (pro) renin receptor attenuates angiotensin-II induced hypertension and renal injury. Circ Res. 2021;129:50–62. 33890822 10.1161/CIRCRESAHA.120.317532 PMC8225587

[43] Fu Z, et al. Mutagenesis of the cleavage site of (pro) renin receptor abrogates aldosterone-salt-induced hypertension and renal injury in mice. Am J Physiol Renal Physiol. 2022. 10.1152/ajprenal.00088.2022 PMC9762973 36302140

[44] Feng Y, Peng K, Luo R, Wang F, Yang T. Site-1 Protease-Derived Soluble (Pro) Renin Receptor Contributes to Angiotensin II–Induced Hypertension in Mice. Hypertension. 2021;77:405–16. 33280408 10.1161/HYPERTENSIONAHA.120.15100 PMC7803453

[45] Wang F, et al. Soluble (pro) renin receptor regulation of ENaC involved in aldosterone signaling in cultured collecting duct cells. Am J Physiol Renal Physiol. 2020;318:F817–25. 31841392 10.1152/ajprenal.00436.2019 PMC7099505

[46] Ramkumar N, et al. Renal tubular epithelial cell prorenin receptor regulates blood pressure and sodium transport. Am J Physiol Renal Physiol. 2016;311:F186–94. 27053687 10.1152/ajprenal.00088.2016 PMC4967157

[47] Delforce SJ, Lumbers ER, Morosin SK, Wang Y, Pringle KG. The Angiotensin II type 1 receptor mediates the effects of low oxygen on early placental angiogenesis. Placenta. 2019;75:54–61. 30712667 10.1016/j.placenta.2018.12.001

[48] Kawai T, et al. AT1 receptor signaling pathways in the cardiovascular system. Pharmacol Res. 2017;125:4–13. 28527699 10.1016/j.phrs.2017.05.008 PMC5607088

[49] Sharma J, Al-Omran A, Parvathy S. Role of nitric oxide in inflammatory diseases. Inflammopharmacology. 2007;15:252–9. 18236016 10.1007/s10787-007-0013-x

[50] Itskovitz J, Sealey JE, Glorioso N, Rosenwaks Z. Plasma prorenin response to human chorionic gonadotropin in ovarian-hyperstimulated women: correlation with the number of ovarian follicles and steroid hormone concentrations. Proc Natl Acad Sci U S A. 1987;84:7285–9. 3118364 10.1073/pnas.84.20.7285 PMC299277

[51] Downing GJ, Maulik D, Poisner AM. Human chorionic gonadotropin stimulates placental prorenin secretion: evidence for autocrine/paracrine regulation. J Clin Endocrinol Metab. 1996;81:1027–30. 8772570 10.1210/jcem.81.3.8772570

[52] Pringle K, Tadros M, Callister R, Lumbers E. The expression and localization of the human placental prorenin/renin-angiotensin system throughout pregnancy: roles in trophoblast invasion and angiogenesis? Placenta. 2011;32:956–62. 22018415 10.1016/j.placenta.2011.09.020

[53] Anton L, et al. Activation of local chorionic villi angiotensin II levels but not angiotensin (1–7) in preeclampsia. Hypertension. 2008;51:1066–72. 18259034 10.1161/HYPERTENSIONAHA.107.103861 PMC2705753

[54] Tamanna S, et al. Renin-angiotensin system (RAS) enzymes and placental trophoblast syncytialisation. Mol Cell Endocrinol. 2022;547: 111609. 35202745 10.1016/j.mce.2022.111609

[55] Morosin SK, Delforce SJ, Lumbers ER, Pringle KG. The (pro) renin receptor (ATP6AP2) does not play a role in syncytialisation of term human primary trophoblast cells. Placenta. 2020;97:89–94. 32792070 10.1016/j.placenta.2020.05.009

[56] Nonn O, et al. Maternal angiotensin increases placental leptin in early gestation via an alternative renin-angiotensin system pathway: suggesting a link to preeclampsia. Hypertension. 2021;77:1723–36. 33775117 10.1161/HYPERTENSIONAHA.120.16425

[57] Weiß E, et al. Maternal overweight downregulates MME (neprilysin) in feto-placental endothelial cells and in cord blood. Int J Mol Sci. 2020;21:834. 32012940 10.3390/ijms21030834 PMC7037888

[58] Williams P, Mistry H, Innes B, Bulmer J, Pipkin FB. Expression of AT1R, AT2R and AT4R and their roles in extravillous trophoblast invasion in the human. Placenta. 2010;31:448–55. 20304486 10.1016/j.placenta.2010.02.014

[59] Li X, et al. Cellular localization of AT1 receptor mRNA and protein in normal placenta and its reduced expression in intrauterine growth restriction. Angiotensin II stimulates the release of vasorelaxants. J Clin Invest. 1998;101:442–54. 9435317 10.1172/JCI119881 PMC508584

[60] Morosin SK, Delforce SJ, Lumbers ER, Pringle KG. Cleavage of the soluble (pro) renin receptor (sATP6AP2) in the placenta. Placenta. 2020;101:49–56. 32920451 10.1016/j.placenta.2020.08.019

[61] Bokuda K, Ichihara A. Possible contribution of (pro)renin receptor to development of gestational diabetes mellitus. World J Diabetes. 2014;5:912–6. 25512796 10.4239/wjd.v5.i6.912 PMC4265880

[62] Almeida LF, Tofteng SS, Madsen K, Jensen BL. Role of the renin–angiotensin system in kidney development and programming of adult blood pressure. Clin Sci. 2020;134:641–56. 10.1042/CS20190765 32219345

[63] Schofield LG, et al*.* Placental deficiency of the (pro) renin receptor ((P) RR) reduces placental development and functional capacity. Front Cell Dev Biol. 11:1212898. 10.3389/fcell.2023.1212898 PMC10427116 37588662

[64] Tower C, et al. Differential expression of angiotensin II type 1 and type 2 receptors at the maternal-fetal interface: potential roles in early placental development. Reproduction. 2010;140:931. 20837730 10.1530/REP-10-0307

[65] Araki-Taguchi M, et al. Angiotensin II mimics the hypoxic effect on regulating trophoblast proliferation and differentiation in human placental explant cultures. Life Sci. 2008;82:59–67. 18048061 10.1016/j.lfs.2007.10.017

[66] Squires PM, Kennedy TG. Evidence for a role for a uterine renin–angiotensin system in decidualization in rats. Reproduction. 1992;95:791–802. 10.1530/jrf.0.0950791 1328628

[67] Walther T, Jank A, Heringer-Walther S, Horn LC, Stepan H. Angiotensin II Type 1 Receptor Has Impact on Murine Placentation. Placenta. 2008;29:905–9. 18722658 10.1016/j.placenta.2008.07.006

[68] Many A, Hubel C, Roberts J. Hyperuricemia and xanthine oxidase in preeclampsia, revisited. Am J Obstet Gynecol. 1996;174:288–91. 8572024 10.1016/s0002-9378(96)70410-6

[69] Stanek J. Histological features of shallow placental implantation unify early-onset and late-onset preeclampsia [published online October 9, 2018]. Pediatr Dev Pathol*. *10. 10.1177/1093526618803759 30301442

[70] Aouache R, Biquard L, Vaiman D, Miralles F. Oxidative stress in preeclampsia and placental diseases. Int J Mol Sci. 2018;19:1496. 29772777 10.3390/ijms19051496 PMC5983711

[71] Mistry H, Kurlak L, Pipkin FB. The placental renin–angiotensin system and oxidative stress in pre-eclampsia. Placenta. 2013;34:182–6. 23246097 10.1016/j.placenta.2012.11.027

[72] Higuchi S, et al. Angiotensin II signal transduction through the AT1 receptor: novel insights into mechanisms and pathophysiology. Clin Sci. 2007;112:417–28. 10.1042/CS20060342 17346243

[73] Herse F, Dechend R, Harsem NK, Wallukat G, Janke J, Qadri F, Hering L, Muller DN, Luft FC, Staff AC. Dysregulation of the circulating and tissue-based renin-angiotensin system in preeclampsia. Hypertension. 2007;49:604–11. 17261642 10.1161/01.HYP.0000257797.49289.71

[74] Shah DM, Banu JM, Chirgwin JM, Tekmal RR. Reproductive tissue renin gene expression in preeclampsia. Hypertens Pregnancy. 2000;19:341–51. 11118408 10.1081/prg-100101996

[75] Conrad KP. Maternal vasodilation in pregnancy: the emerging role of relaxin. Am J Physiol Regul Integr Comp Physiol. 2011;301:R267–75. 21613576 10.1152/ajpregu.00156.2011 PMC3154715

[76] Derkx F, et al. Source of plasma prorenin in early and late pregnancy: observations in a patient with primary ovarian failure. J Clin Endocrinol Metab. 1987;65:349–54. 2439529 10.1210/jcem-65-2-349

[77] Merrill DC, Karoly M, Chen K, Ferrario CM, Brosnihan KB. Angiotensin-(1–7) in normal and preeclamptic pregnancy. Endocrine. 2002;18:239–45. 12450315 10.1385/ENDO:18:3:239

[78] Hsueh W, et al. Changes in active and inactive renin throughout pregnancy. J Clin Endocrinol Metab. 1982;54:1010–6. 7037818 10.1210/jcem-54-5-1010

[79] Brosnihan K, et al. Enhanced expression of Ang-(1–7) during pregnancy. Braz J Med Biol Res. 2004;37:1255–62. 15273828 10.1590/s0100-879x2004000800017

[80] Schefe JH, et al. A novel signal transduction cascade involving direct physical interaction of the renin/prorenin receptor with the transcription factor promyelocytic zinc finger protein. Circ Res. 2006;99:1355–66. 17082479 10.1161/01.RES.0000251700.00994.0d

[81] Schefe JH, et al. Prorenin engages the (pro) renin receptor like renin and both ligand activities are unopposed by aliskiren. J Hypertens. 2008;26:1787–94. 18698213 10.1097/HJH.0b013e3283060f2e

[82] Lumbers ER. Peripheral vascular reactivity to angiotensin and noradrenaline in pregnant and non-pregnant women. Aust J Exp Biol Med Sci. 1970;48:493–500. 4321323 10.1038/icb.1970.49

[83] Gant NF, Daley GL, Chand S, Whalley PJ, MacDonald PC. A study of angiotensin II pressor response throughout primigravid pregnancy. J Clin Investig. 1973;52:2682–9. 4355997 10.1172/JCI107462 PMC302534

[84] Neves LA, et al. Pregnancy enhances the angiotensin (Ang)-(1–7) vasodilator response in mesenteric arteries and increases the renal concentration and urinary excretion of Ang-(1–7). Endocrinology. 2003;144:3338–43. 12865311 10.1210/en.2003-0009

[85] Watanabe N, et al. Soluble (pro) renin receptor and blood pressure during pregnancy: a prospective cohort study. Hypertension. 2012;60:1250–6. 23045457 10.1161/HYPERTENSIONAHA.112.197418

[86] Thomason J, et al. Elevation of (pro) renin and (pro) renin receptor in preeclampsia. Am J Hypertens. 2015;28:1277–84. 25767135 10.1093/ajh/hpv019

[87] Sugulle M, et al. Soluble (pro) renin receptor in preeclampsia and diabetic pregnancies. J Am Soc Hypertens. 2017;11:644–52. 29050747 10.1016/j.jash.2017.08.001

[88] Ohwaki A, et al. Altered serum soluble furin and prorenin receptor levels in pregnancies with pre-eclampsia and fetal growth restriction. J Gynecol Obstet Hum Reprod. 2021;50. 34289413 10.1016/j.jogoh.2021.102198

[89] Schofield LG, et al. The soluble (pro) renin receptor promotes a preeclampsia-like phenotype both in vitro and in vivo. Hypertens Res. 2024;1–15. 10.1038/s41440-024-01678-8 PMC11150152 38605139

[90] Verdonk K, Visser W, Van Den Meiracker AH, Danser AJ. The renin–angiotensin–aldosterone system in pre-eclampsia: the delicate balance between good and bad. Clin Sci. 2014;126:537–44. 10.1042/CS20130455 24400721

[91] Zhou A, et al. A redox switch in angiotensinogen modulates angiotensin release. Nature. 2010;468:108–11. 20927107 10.1038/nature09505 PMC3024006

[92] AbdAlla S, Lother H, el Massiery A, Quitterer U. Increased AT 1 receptor heterodimers in preeclampsia mediate enhanced angiotensin II responsiveness. Nat Med. 2001;7:1003–9. 11533702 10.1038/nm0901-1003

[93] Quitterer U, Lother H, Abdalla S. AT1 receptor heterodimers and angiotensin II responsiveness in preeclampsia. in Seminars in nephrology, Vol. 24 115–119 (Elsevier, 2004). 10.1016/j.semnephrol.2003.11.007 15017523

[94] AbdAlla S, Abdel-Baset A, Lother H, El Massiery A, Quitterer U. Mesangial AT 1/B 2 receptor heterodimers contribute to angiotensin II hyperresponsiveness in experimental hypertension. J Mol Neurosci. 2005;26:185–92. 16012192 10.1385/JMN:26:2-3:185

[95] Xia Y, Kellems RE. Angiotensin receptor agonistic autoantibodies and hypertension: preeclampsia and beyond. Circ Res. 2013;113:78–87. 23788505 10.1161/CIRCRESAHA.113.300752 PMC4131731

[96] Xia Y, Kellems RE. Receptor-activating autoantibodies and disease: preeclampsia and beyond. Expert Rev Clin Immunol. 2011;7:659–74. 21895478 10.1586/eci.11.56 PMC3268148

[97] LaMarca B, Wallace K, Granger J. Role of angiotensin II type I receptor agonistic autoantibodies (AT1-AA) in preeclampsia. Curr Opin Pharmacol. 2011;11:175–9. 21317038 10.1016/j.coph.2011.01.003 PMC3075337

[98] Navar LG. Intrarenal renin-angiotensin system in regulation of glomerular function. Curr Opin Nephrol Hypertens. 2014;23:38. 24275770 10.1097/01.mnh.0000436544.86508.f1 PMC3982859

[99] Sequeira-Lopez MLS, Gomez RA. Renin cells, the kidney, and hypertension. Circ Res. 2021;128:887–907. 33793334 10.1161/CIRCRESAHA.121.318064 PMC8023763

[100] Schnermann J. Cyclooxygenase-2 and macula densa control of renin secretion. Nephrol Dial Transplant. 2001;16:1735–8. 11522847 10.1093/ndt/16.9.1735

[101] Shao W, Seth DM, Navar LG. Augmentation of endogenous intrarenal angiotensin II levels in Val5-ANG II-infused rats. Am J Physiol Renal Physiol. 2009;296:F1067–71. 19244403 10.1152/ajprenal.90596.2008 PMC2681365

[102] Tanabe A, et al. Angiotensin II stimulates both aldosterone secretion and DNA synthesis via type 1 but not type 2 receptors in bovine adrenocortical cells. J Endocrinol Invest. 1998;21:668–72. 9854682 10.1007/BF03350796

[103] Matsukawa T, Miyamoto T. Angiotensin II-stimulated secretion of arginine vasopressin is inhibited by atrial natriuretic peptide in humans. Am J Physiol Regul Integr Comp Physiol. 2011;300:R624–9. 21123762 10.1152/ajpregu.00324.2010

[104] McKinley MJ, et al. Physiological and pathophysiological influences on thirst. Physiol Behav. 2004;81:795–803. 15234185 10.1016/j.physbeh.2004.04.055

[105] Ramchandra R, Yao ST, May CN. Organ selective regulation of sympathetic outflow by the brain angiotensin system. Curr Hypertens Rep. 2013;15:401–8. 23681510 10.1007/s11906-013-0355-2

[106] Fu Z, Hu J. (Pro)renin receptor contributes to pregnancy-induced sodium-water retention in rats via activation of intrarenal RAAS and -ENaC. Am J Physiol Renal Physiol. 2019;316:F530–8. 30379098 10.1152/ajprenal.00411.2018 PMC6459302

[107] Zou L-X, et al. Receptor-mediated intrarenal angiotensin II augmentation in angiotensin II–infused rats. Hypertension. 1996;28:669–77. 8843896 10.1161/01.hyp.28.4.669

[108] Nishiyama A, Seth DM, Navar LG. Renal interstitial fluid concentrations of angiotensins I and II in anesthetized rats. Hypertension. 2002;39:129–34. 11799091 10.1161/hy0102.100536

[109] Wang Y, et al. (Pro) renin receptor antagonist PRO20 attenuates nephrectomy-induced nephropathy in rats via inhibition of intrarenal RAS and Wnt/β-catenin signaling. Physiol Rep. 2021;9: e14881. 34057312 10.14814/phy2.14881 PMC8165733

[110] Velauthar L, et al. First-trimester uterine artery Doppler and adverse pregnancy outcome: a meta-analysis involving 55 974 women. Ultrasound Obstet Gynecol. 2014;43:500–7. 24339044 10.1002/uog.13275

[111] O’Gorman N, et al. Accuracy of competing-risks model in screening for pre-eclampsia by maternal factors and biomarkers at 11–13 weeks’ gestation. Ultrasound Obstet Gynecol. 2017;49:751–5. 28067011 10.1002/uog.17399

[112] Zeisler H, et al. Predictive value of the sFlt-1: PlGF ratio in women with suspected preeclampsia. N Engl J Med. 2016;374:13–22. 26735990 10.1056/NEJMoa1414838

[113] Leaños-Miranda A, et al. Changes in circulating concentrations of soluble fms-like tyrosine kinase-1 and placental growth factor measured by automated electrochemiluminescence immunoassays methods are predictors of preeclampsia. J Hypertens. 2012;30:2173–81. 22902831 10.1097/HJH.0b013e328357c0c9

[114] Roberts JM, Escudero C. The placenta in preeclampsia. Pregnancy Hypertension: An International Journal of Women’s Cardiovascular Health. 2012;2:72–83. 10.1016/j.preghy.2012.01.001 PMC3381433 22745921

[115] Atallah A, et al. Aspirin for prevention of preeclampsia. Drugs. 2017;77:1819–31. 29039130 10.1007/s40265-017-0823-0 PMC5681618

[116] Sorkin E, Clissold S, Brogden R. Nifedipine Drugs. 1985;30:182–274. 2412780 10.2165/00003495-198530030-00002

[117] Goa KL, Benfield P, Sorkin EM. Labetalol Drugs. 1989;37:583–627. 2663413 10.2165/00003495-198937050-00002

[118] Mah GT, Tejani AM, Musini VM. Methyldopa for primary hypertension. Cochrane Database Syst Rev. 2009. 10.1002/14651858.CD003893.pub3 PMC7154320 19821316

[119] Odigboegwu O, Pan LJ, Chatterjee P. Use of antihypertensive drugs during preeclampsia. Frontiers in cardiovascular medicine. 2018;5:50. 29896480 10.3389/fcvm.2018.00050 PMC5987086

[120] Dasgupta S, Sarkhel A, Jain A. Single loading dose of magnesium sulphate in severe preeclampsia and eclampsia-is it effective? A randomized prospective study. Obstet Gynecol Int J. 2015;2:59.

[121] Li X, et al. An analysis of the differences between early and late preeclampsia with severe hypertension. Pregnancy Hypertension: An International Journal of Women’s Cardiovascular Health. 2016;6:47–52. 10.1016/j.preghy.2015.12.003 26955772

[122] Guron G, Friberg P. An intact renin–angiotensin system is a prerequisite for normal renal development. J Hypertens. 2000;18:123–37. 10694179 10.1097/00004872-200018020-00001

[123] Hanssens M, Keirse M, Vankelecom F, Van Assche FA. Fetal and neonatal effects of treatment with angiotensin-converting enzyme inhibitors in pregnancy. Obstet Gynecol. 1991;78:128–35. 2047053

[124] Seely EW, Ecker J. Chronic hypertension in pregnancy. Circulation. 2014;129:1254–61. 24637432 10.1161/CIRCULATIONAHA.113.003904

[125] Zhang B, et al. Placenta-specific drug delivery by trophoblast-targeted nanoparticles in mice. Theranostics. 2018;8:2765. 29774074 10.7150/thno.22904 PMC5957008

[126] Chappell MC. S1P (Site-1 Protease)-Induced Release of the Soluble Prorenin Receptor in Hypertension: Do All Roads Lead to Ang II (Angiotensin II)? Hypertension. 2021;77:417–9. 33439731 10.1161/HYPERTENSIONAHA.120.16428 PMC7810158

[127] Couture F, D'Anjou F, Day R. On the cutting edge of proprotein convertase pharmacology: from molecular concepts to clinical applications. 2011. 10.1515/bmc.2011.034 PMC3270943 22308173

[128] Jean F, et al. α1-Antitrypsin Portland, a bioengineered serpin highly selective for furin: application as an antipathogenic agent. Proc Natl Acad Sci. 1998;95:7293–8. 9636142 10.1073/pnas.95.13.7293 PMC22594

[129] Li W, et al. Intracerebroventricular infusion of the (pro) renin receptor antagonist PRO20 attenuates deoxycorticosterone acetate-salt–induced hypertension. Hypertension. 2015;65:352–61. 25421983 10.1161/HYPERTENSIONAHA.114.04458 PMC4902274

[130] Mishima S, et al. Endothelin-1 production via placental (pro)renin receptor in a mouse model of preeclampsia. Placenta. 2023;138:44–50. 37167782 10.1016/j.placenta.2023.05.002

[131] Mishima S, et al. Elucidation of blood pressure elevation mechanism mediated by placental (pro)renin receptors in preeclampsia model mice. Placenta. 2023;140:e79.

